# Brain connectivity aberrations in anabolic-androgenic steroid users

**DOI:** 10.1016/j.nicl.2016.11.014

**Published:** 2016-11-17

**Authors:** Lars T. Westlye, Tobias Kaufmann, Dag Alnæs, Ingunn R. Hullstein, Astrid Bjørnebekk

**Affiliations:** a NORMENT, KG Jebsen Centre for Psychosis Research, Division of Mental Health and Addiction, Oslo University Hospital & Institute of Clinical Medicine, University of Oslo, Norway; b Department of Psychology, University of Oslo, Norway; c Norwegian Doping Control Laboratory, Oslo University Hospital, Norway; d Division of Mental Health and Addiction, Department on Substance Use Disorder Treatment, Norwegian National Advisory Unit on Substance Use Disorder Treatment, Oslo University Hospital, Norway

**Keywords:** Anabolic-androgenic steroids (AAS), fMRI, Amygdala, Functional connectivity, Default-mode network, Dependency

## Abstract

Sustained anabolic-androgenic steroid (AAS) use has adverse behavioral consequences, including aggression, violence and impulsivity. Candidate mechanisms include disruptions of brain networks with high concentrations of androgen receptors and critically involved in emotional and cognitive regulation. Here, we tested the effects of AAS on resting-state functional brain connectivity in the largest sample of AAS-users to date. We collected resting-state functional magnetic resonance imaging (fMRI) data from 151 males engaged in heavy resistance strength training. 50 users tested positive for AAS based on the testosterone to epitestosterone (T/E) ratio and doping substances in urine. 16 previous users and 59 controls tested negative. We estimated brain network nodes and their time-series using ICA and dual regression and defined connectivity matrices as the between-node partial correlations. In line with the emotional and behavioral consequences of AAS, current users exhibited reduced functional connectivity between key nodes involved in emotional and cognitive regulation, in particular reduced connectivity between the amygdala and default-mode network (DMN) and between the dorsal attention network (DAN) and a frontal node encompassing the superior and inferior frontal gyri (SFG/IFG) and the anterior cingulate cortex (ACC), with further reductions as a function of dependency, lifetime exposure, and cycle state (on/off).

## Highlights

• Sustained AAS use has adverse behavioral consequences, including aggression, violence and impulsivity.
• We obtained r-fMRI data from 50 male users testing positive for AAS and 16 previous users and 59 controls testing negative.
• We used ICA and dual regression, and defined connectivity matrices as the between-node temporal partial correlations.
• Current users showed significantly reduced connectivity between amygdala and DMN and between DAN and a SFG/IFG/ACC node.

## 1 Introduction

Anabolic androgenic steroids (AAS) comprise a large category of synthetic derivatives of the male sex hormone testosterone widely used for cosmetic or ergogenic purposes (Ip et al., 2011, Kanayama et al., 2009b, Kanayama et al., 2001). In addition to the performance enhancing and tissue building properties, AAS is associated with a wide range of symptoms, including aggression, violence and impulsive behaviors (Pagonis et al., 2006b, Trenton and Currier, 2005). While positive effects of AAS on mood, such as transient euphoria and hypomania, have been reported early in the course of AAS use (Thiblin and Petersson, 2005), anxiety, impulsivity, marked irritability and aggression is commonly manifested after long-term use (Hall and Chapman, 2005, Pagonis et al., 2006b, Pope et al., 2000, Trenton and Currier, 2005). Moreover, emerging evidence suggests that prolonged AAS use is associated with cognitive impairments including self-reported memory (Heffernan et al., 2015a, Su et al., 1993), working memory and visuospatial abilities (Kanayama et al., 2012, Kaufman et al., 2015).

Whereas the exact mechanisms of the adverse consequences of AAS use are unclear, they are likely partly reflecting disruptions of brain networks implicated in emotional and cognitive regulation. AAS readily passes the blood-brain barrier and affect central nervous system function. AAS binds to cytoplasmic androgen receptors (Janne et al., 1993), whereby the bound receptor is translocated into the nucleus where it binds to specific response elements in target genes and triggers DNA transcription and protein synthesis (Heinlein and Chang, 2002, Keller et al., 1996). Androgen receptors are abundantly expressed in the amygdala, hippocampus, brain stem, hypothalamus, and cerebral cortex (Kritzer, 2004, Pomerantz et al., 1985, Simerly et al., 1990), implicating a wide range of functions, including regulation of emotion and cognition.

Commonly, AAS is administrated in cycles lasting for 8–16 weeks interspersed with drug-free intervals. During cycles a variety of AAS compounds are usually co-administered known as “stacking” with doses exceeding therapeutic levels by 5–100-fold in males [7–9], thereby generating highly non-physiological levels of endogenous and synthetic testosterone, with adverse effects on brain function (Clark et al., 1995, Oberlander and Henderson, 2012). Whereas some users ingest AAS only a few times during a lifetime, others develop a dependency syndrome, with sustained use despite adverse effects (Kanayama et al., 2009a). The range and severity of the behavioral consequences increase with the severity of abuse (Pagonis et al., 2006a). Exogenous AAS administration suppresses the hypothalamic-pituitary-testicular (HPT) axis, causing decreased endogenous testosterone production in males (39, 40). Cycles are used with the rationale that the HPT axis may recuperate during AAS-free intervals, restoring normal endogenous testosterone production (Reyes-Fuentes and Veldhuis, 1993). Thus, classical AAS administration results in substantial fluctuations of endogenous and synthetic testosterone throughout the cycle. Such hormonal fluctuations likely influence brain functions, and might explain AAS induced alterations in mood and behavior (Pope and Katz, 1994, Su et al., 1993).

Only one previous study has investigated functional brain networks as measured using functional MRI (fMRI) after prolonged AAS use. Using a seed-based approach targeting amygdala connectivity, it was found that resting-state coupling between the right amygdala and frontal, striatal, limbic, hippocampal, and visual cortical areas, respectively, was significantly lower in 7 users compared to 9 non-users (Kaufman et al., 2015). Further, a recent meta-analysis of fMRI activation studies revealed significant amygdala foci both after testosterone administration and in endogenous testosterone studies (Heany et al., 2016), and higher endogenous testosterone levels have been linked to attenuated resting-state amygdala-prefrontal coupling in adolescents (Peters et al., 2015), and in healthy males during a social approach-avoidance task (Volman et al., 2011), and intranasal testosterone reduced amygdala coupling with the orbitofrontal cortex in females (van Wingen et al., 2011).

Supraphysiological doses of AAS may cause apoptotic effects on a variety of cell types including neurons (Basile et al., 2013, Caraci et al., 2011, Cunningham et al., 2009, Estrada et al., 2006, Orlando et al., 2007). In an overlapping sample, prolonged AAS use was associated with smaller gray matter, cortical and putamen volumes and thinner cortex, with stronger effects with increasing exposure, also in users without any other substance abuse (Bjørnebekk et al., 2016). Summarized, converging evidence suggests that prolonged AAS use with supraphysiological doses is associated with both structural and functional brain alterations. However, available neuroimaging studies of AAS users are rare and limited by small samples sizes.

The aim of the current study was to test the effects of sustained AAS use on resting-state functional connectivity in a sample of male long-term AAS users and non-users. After rigorous denoising of the individual datasets to minimize the impact of motion and other artifacts, spatial maps constituting the nodes in the functional brain network and their associated time-series were estimated using spatial group independent component analysis (ICA) and dual regression. We defined the brain connectivity indices as the between-node partial temporal correlations, yielding a node-by-node correlation matrix for each dataset, where each node pair is referred to as an edge in the network. Next, we tested for associations between AAS status (current user, previous user or control) and connectivity strength using edgewise analysis. In order to further characterize the clinical sensitivity, we tested for associations with AAS dependency, lifetime exposure and AAS cycle state (on vs. off). Since elevated testosterone to epitestosterone (T/E) ratio may indicate use of testosterone, we tested for associations between connectivity and T/E ratios in the full sample (n = 142).

Based on existing evidence reviewed above, we anticipated that discriminative connections would implicate regions according to the anatomical distribution of androgen receptors, including the amygdala, hippocampus, and brain stem, and their synchronization with cortical nodes (Kritzer, 2004, Pomerantz et al., 1985, Simerly et al., 1990). In particular, due to the characteristic emotional consequences of AAS use, and converging neuroimaging evidence (Kaufman et al., 2015, Peters et al., 2015, van Wingen et al., 2011, Volman et al., 2011), we expected group differences in amygdala connectivity and in brain network nodes involved in cognitive and emotional regulation, which would also be sensitive to the severity of dependence and total exposure.

## 2 Materials and methods

### 2.1 Subjects

Table 1 summarizes demographic and clinical characteristics of the sample. Males engaged in heavy resistance strength training who were either current or previous AAS users reporting at least one year of cumulative AAS exposure (summarizing on-cycle periods) or who had never tried AAS or equivalent doping substances were recruited through webpages and forums targeting people interested in heavy weight training, bodybuilding, and online forums (open and closed) directly addressing steroid use. In addition, posters and flyers were distributed on select gyms in Oslo. In the recruitment information the study aim was explicitly stated. Prior to enrollment all participants received an information brochure with a complete description of the study. The study was approved by the Regional Committees for Medical and Health Research Ethics South East Norway (REC) (2013/601), all research was carried out in accordance with the Declaration of Helsinki, and written informed consent was collected from all subjects. Participants were compensated with 1000 NOK (approx. 125 USD). The sample is partly overlapping with the one described in detail in Bjørnebekk et al. (Bjørnebekk et al., 2016). Here, we were primarily interested in brain connectivity alterations related to AAS-induced hormonal fluctuations. Thus, for the group analyses participants were divided into current, previous or a control group that in addition to self-reports were confirmed by the doping analyses. Participants failing to meet these strict group definitions were excluded from the group analyses, but were included in analyses testing for associations with T/E ratio across groups.

Resting-state fMRI data was obtained from 151 individuals, including 82 previous or current AAS users and 69 non-users based on self-reports. Of the 69 non-using *controls* one was excluded due to neuroradiological findings, one because he failed to match criteria for strength training (see Bjørnebekk et al., 2016 for details). In addition, three participants were excluded due to missing urine samples and five for having T/E ratios > 4 (range 4.3–8.4), which might indicate administration of testosterone, yielding 59 participants in this group. Among the 82 AAS users with rFMRI data, two subjects were excluded due to less than a year of accumulated use, and two due to missing urine sample. The remaining 78 datasets comprised 58 *current users* and 20 *previous users*. Among the 58 *current users* reporting AAS use within the past 12 months eight were excluded due to a negative AAS test, yielding 50 current users. Among the 20 *previous users* with a self-reported history of AAS use in the past (> 12 months prior to scanning) four were excluded due to traces of AAS or testosterone use in the urine, yielding 16 previous users. Supplemental Table 1 provides group summary stats and comparison on use characteristics for current, previous and excluded users. Material and methods used to assess AAS use, medical history, AAS dependence, verbal IQ, alcohol and drug use, mood and problem behavior are summarized in the Supplement.

### 2.2 Doping analysis

Urine samples were collected at the same visit as the cognitive testing and analyzed for AAS and narcotics by gas chromatography and mass spectrometry at the WADA accredited Norwegian Doping Laboratory at Oslo University Hospital (Hullstein et al., 2015). Stimulants were analyzed by liquid chromatography and mass spectrometry.

Briefly, the criteria used to determine the use of AAS or testosterone are 1) urine samples positive for AAS compounds 2) a T/E ratio > 4 as has been applied by World Anti-Doping Agency as a population based criteria for samples requiring further analysis by isotope ratio mass spectrometry (IRMS) or follow up to indicate testosterone abuse (group, 2016). When applying this criterion in research and routine analyses, cases of naturally occurring T/E ratios above 4 appear (Mareck et al., 2010), and sometimes a stricter T/E ratio is preferred (Hullstein et al., 2015). Supplemental Fig. 1 and Supplemental Table 2 provides a summary of the frequency of the specific anabolic-androgenic steroids found in the urine sample, and Supplemental Table 3 summarizes the most popular compounds based on self-reports.

### 2.3 MRI acquisition

MRI scans were obtained on a Siemens Skyra 3T scanner with a 24-channel head coil at Oslo University Hospital. We acquired structural MRI with a T1-weighted 3D magnetization-prepared rapid gradient-echo (MPRAGE) sequence, with the following parameters (TR: 2300 ms; TE: 2.98 ms; FA: 8°; voxel size: 1 × 1 × 1 mm; 176 sagittal slices) and fMRI data with a T2*-weighted 2D gradient echo-planar imaging sequence (EPI) with 150 volumes (TR: 2390 ms; TE: 30 ms; FA: 90°; voxel size: 3 × 3 × 3 mm; 43 axial slices). Participants were instructed to keep their eyes open.

### 2.4 MRI processing and network estimation

For fMRI analysis we used the FMRI Expert Analysis Tool (FEAT) from the FMRIB Software Library (FSL) (Smith et al., 2004). The pipeline included motion correction (MCFLIRT), spatial smoothing (full width at half maximum of 6 mm), grand-mean intensity normalisation of the entire 4D dataset by a single multiplicative factor, high-pass temporal filtering (Gaussian-weighted least-squares straight line fitting, sigma = 45 s), and single-session independent component analysis (ICA) using MELODIC (Beckmann and Smith, 2004)

We used FIX (ICA-based Xnoisefier (Salimi-Khorshidi et al., 2014)), to identify and remove noise components on an individual level using a machine learning approach with a standard training set (threshold: 20). FIX was recently shown to outperform several different methods for data cleaning (Pruim et al., 2015)). Supplemental Fig. 2 shows average temporal signal-to-noise ratio (tSNR) (Roalf et al., 2016) before and after FIX, and details regarding group differences are reported in the Supplement.

We extracted brain masks from the T1-weighted volumes using Freesurfer (Fischl et al., 2002), used for registration to standard space using FLIRT (Jenkinson and Smith, 2001) with boundary-based registration (BBR, (Greve and Fischl, 2009)) and FNIRT (Andersson et al., 2010).

After registration, we employed group independent component analysis using MELODIC including all datasets by mean of a group-PCA technique (Smith et al., 2014). Automated model order selection yielded 47 components. Next, for each subject we estimated individual time series and component spatial maps using dual regression (Filippini et al., 2009). Per recommendations (Kelly et al., 2010), based on the component spatial maps and the frequency spectrum of the time series, we identified six noise components and regressed the time-series from these out of the remaining components. Next, we excluded another 11 components of which spatial maps were not corresponding to any interpretable neuronal origin or were outside the mask. The remaining 30 components constituted the nodes in subsequent network analyses, and the corresponding 435 temporal partial correlations between each component pairs formed the edges (connections) of the full network. As in previous studies (Kaufmann et al., 2016, Kaufmann et al., 2015, Skatun et al., n.d), for each subject, we computed the regularized partial correlations (Smith et al., 2011) with automated individual estimation of regularization strength (Ledoit and Wolf, 2003).

### 2.5 Statistical analysis

We assessed effects of AAS use on between-node connectivity by comparing current users, previous users and controls using analysis of covariance (ANCOVA) on each network edge, covarying for age. We corrected for multiple comparisons using Bonferroni correction across all 435 edges (adjusted alpha = 0.05/435). Next, in any edges showing significant effects of group, we tested for associations with dependency, life time exposure, and cycle state using ANCOVAs covarying for age (see Supplemental Information for details). To assess the relative importance of each node in distinguishing between groups, we calculated the eigenvector centrality of each node based on the edge-wise F-values from the group ANCOVA. A high centrality indicates altered connectivity with several other nodes, indicating a relative importance of this node in discriminating between groups, regardless of the significance threshold applied on the edge level.

To control for the possible influence of general cognitive function, aspects of mental health, alcohol and drug use on between-node connectivity, we conducted additional analyses where IQ, weekly alcohol consumption and the ASEBA ASR T-scores for anxious/depressed syndrome, drug use, attention problem and total problems were included as additional covariates (one at a time). Also, we conducted analyses where participants with traces of narcotics in the urine were excluded, in order to reassure that the findings were not a result of the use of narcotics. Finally, we assessed the relations between T/E ratio and edge strengths by Spearman's Rho correlations (T/E ratio distribution was positively skewed) across the full sample (n = 142).

## 3 Results

### 3.1 Edgewise connectivity

**Fig. 1:**
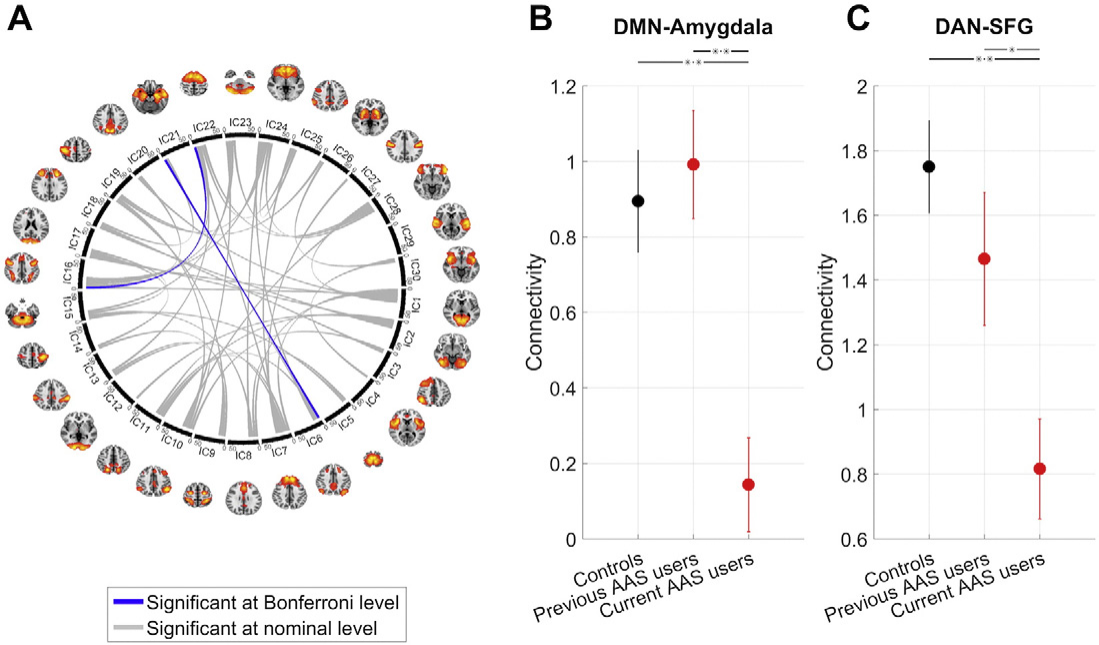
Results from the edgewise ANCOVA testing for differences between current AAS users, previous AAS users and controls. Fig. 1A: 49 edges showed nominal (p < 0.05, uncorrected) and two edges showed significant (p < 0.05, Bonferroni corrected) group effects, indicating reduced connectivity in links between the DMN and the amygdala, and between the DAN and SFG/IFG/ACC. Fig. 1B–C shows the mean connectivity within each of the groups for the two significant edges. Error bars denote standard error of the means (SEM).

Fig. 1A shows the results from the edgewise ANCOVA testing for differences between current AAS users (n = 50), previous AAS users (n = 16) and controls (n = 59). 49 (11.3%) of the edges showed nominal (p < 0.05, uncorrected) and two edges showed significant (p < 0.05, Bonferroni corrected) group effects. The two significant edges comprised the connections between the default mode network (DMN) and the amygdala (F = 10.2, p < 8e − 05), and between the dorsal attention network (DAN) and a node encompassing the superior and inferior frontal gyrus (SFG/IFG) and the anterior portions of the anterior cingulate cortex (ACC) (F 9.8, p < 1e − 04), both indicating reductions in functional connectivity in current AAS users compared to both non-users (t > 4.0, p < .1e − 04) and previous users (t > 2.1, p < 0.03), yet no difference between previous users and controls (t < 0.97, p > 0.33). Fig. 1B–C illustrates the corresponding connectivity patterns for both edges. The findings were replicated in analyses excluding participants with traces of narcotics in the urine, or with concurrent substance abuse. Including IQ, drug use, alcohol consumption, symptoms of anxiety and depression, attention problems and ASR total problem scores in the statistical models only marginally influenced the findings (Supplemental Table 4).

Supplemental Fig. 3 shows the EC value per node, reflecting a nodewise summary measure of effect size on tests for differences between current users, previous users and controls. The nodes showing strongest overall centrality were IC22 (SFG/IFG/ACC) and IC16 (DAN).

**Fig. 2:**
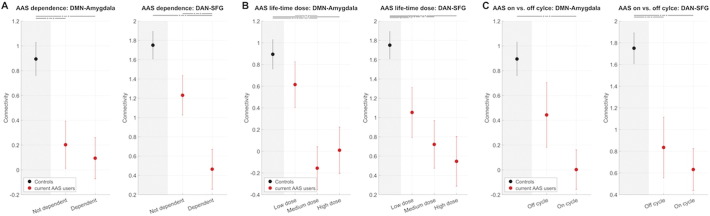
Edge connectivity stratified by clinical information. Fig. 2A: Mean connectivity for current AAS users with and without dependence. Fig. 2B: Mean connectivity for current AAS users by lifetime exposure (low dose, medium dose, high dose). Fig. 2C: Mean connectivity strength for current AAS users by cycle status (on/off). Error bars denote standard errors of the mean (SEM).

Fig. 2A splits current AAS users into individuals with (n = 27) and without (n = 23) dependence. Whereas the difference between dependent and non-dependent users was not significant in the DMN-amygdala edge, there was a clear pattern of controls > non-dependent users > dependent users in the DAN-SFG edge, with dependent users significantly different both from non-dependents users (t = 2.6, p = 0.01) and controls (t = 5.1, p = 2e − 06).

Fig. 2B splits the current AAS users according to their lifetime exposure (low dose: n = 15, medium dose: n = 17, high dose: n = 17). Both edges revealed a clear dose effect. There was a nominal significant difference between low dose and medium dose in the DMN-amygdala edge (t = 2.7, p = 0.013) and close to significance for low vs. high dose (t = 2.0, p = 0.054). Controls were significantly different from medium and high dose lifetime exposure groups. The DAN-SFG edge revealed a striking connectivity pattern of low dose > medium dose > high dose, although only differences between controls and any of the dose groups were significant.

Fig. 2C splits current users into those currently off cycle (n = 15) and those currently on cycle (n = 29), revealing a pattern of off cycle > on cycle in mean connectivity. For the DMN-amygdala edge, controls significantly differed from current users on cycle (t = 4.0, p = 1e − 04) but not off cycle (t = 1.5, p = 0.13). For the DAN-SGF edge, controls differed from users on (t = 4.6, p = 1e − 05) and off cycle (t = 2.9, p = 0.005).

**Fig. 3:**
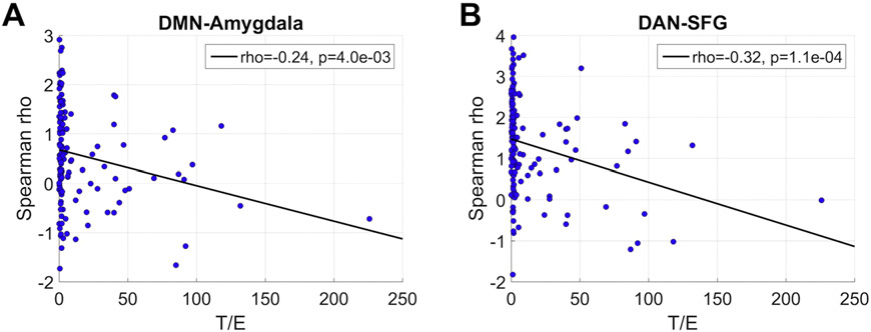
Edge connectivity plotted as a function of T/E ratio across the full sample (n = 142).

Finally, we assessed associations between T/E ratio and connectivity in the two main edges across the full sample (n = 142), independent of group assignment. Fig. 3A–B reveals decreased connectivity with increasing T/E, both for DMN-amygdala (rho = − 0.24, p = 4e − 03) and DAN-SFG (rho = − 0.32, p = 1e − 04). Supplemental Fig. 4 illustrates that the negative association persists across the full T/E span.

### 3.2 tSNR and motion as potential confounders

In addition to denoising individual fMRI datasets using FIX (Salimi-Khorshidi et al., 2014), we tested if effects of AAS remained when including tSNR and motion as additional covariates in the statistical models. Briefly, whereas effect sizes were somewhat reduced, both edges still showed significant group effects (DMN-amygdala: F = 6.8, p = 0.002, DAN-SFG: 6.6, p = 0.001) when accounting for tSNR and motion.

## 4 Discussion

In the largest functional neuroimaging study of current and previous AAS users to date, we have demonstrated robust functional brain connectivity reductions between major brain hubs modulating emotional and cognitive functions. Specifically, we have documented reduced connectivity between the DMN and the amygdala and between the DAN and a frontal node encompassing the SFG, IFG and the ACC in current users compared to non-users and previous users. The connectivity was further reduced in dependent users or those on cycle, showed a graded pattern of reductions when comparing subjects with low, medium and high lifespan exposure, and was negatively correlated with T/E ratio across the full sample.

Both theoretical considerations and empirical evidence suggest involvement of the amygdala as a hub mediating the adverse behavioral and emotional effects of AAS. However, sample sizes have been limited, which challenges the reliability of previous reports. Therefore, we applied a data driven approach, allowing detection of additional hubs beyond the amygdala, and assessment of anatomical specificity. In line with our hypotheses, we observed amygdala connectivity reduction in users, and further reductions with increased lifetime use and T/E ratio.

Despite the use of methods that are not necessarily directly comparable, our data-driven approach supports evidence from a small-scale study suggesting decreased functional connectivity between the amygdala and other brain regions in AAS users versus non-users (Kaufman et al., 2015). The findings are also in accordance with previous fMRI reports linking increases in testosterone levels to reductions in amygdala-prefrontal coupling (Peters et al., 2015, Spielberg et al., 2015, Volman et al., 2011), and reinforces the notion that testosterone fluctuations affect networks in the brain which are critically involved in emotional processing and regulation (Li et al., 2014, Phelps and LeDoux, 2005).

The AAS effects on amygdala coupling are compatible with amygdala being among the brain regions with the highest androgen receptor mRNA density (Menard and Harlan, 1993, Michael et al., 1995, Simerly et al., 1990). AAS has been associated with a wide array of adverse effects on mental health and also cognitive deficits (Heffernan et al., 2015b, Kanayama et al., 2012, Kaufman et al., 2015, Su et al., 1993). Perturbations of amygdala connectivity with cognitive and emotional network hubs including the DMN could potentially constitute brain correlates of these side effects.

In an overlapping sample it was recently reported smaller neuroanatomical volumes in AAS-users compared to non-users, including overall cortical, total gray matter, corpus callosum and putamen volumes and thinner cortex in widespread regions, including the posterior cingulate cortices, which are key regions in the DMN (Bjørnebekk et al., 2016). Whereas DMN connectivity has been implicated in a range of psychiatric and neurological conditions (Buckner et al., 2008, Whitfield-Gabrieli and Ford, 2012), suggesting that DMN connectivity is a sensitive but not specific index of brain function, our findings of reduced DMN-amygdala connectivity are in line with extant evidence of emotional dysregulation in AAS users.

The other edge showing reduced connectivity in AAS users implicated the DAN and the SFG/IFG/ACC. DAN, comprising key hubs in the intraparietal sulcus (IPS), superior parietal lobule (SPL), and the frontal eye field (Corbetta et al., 2008), constitutes a core attention node. The connectivity patterns of the DAN are predictive of cognitive load during a multiple object tracking (Alnaes et al., 2015), which supports its sensitivity to mental states associated with goal-driven attention. SFG constitutes a collection of distinct subregions in the prefrontal cortex, which has been implicated in a wide range of executive functions, including working memory and cognitive control (du Boisgueheneuc et al., 2006, Fuster, 2001). The structural connectivity patterns of the anteromedial part of the SFG, which showed strongest loading in the implicated component, have been involved in response selection and cognitive and attentional control (Li et al., 2013). The right IFG has consistently been implicated in response inhibition and impulse control (Aron et al., 2014), and damage of the right IFG are detrimental for stop-signal inhibition (Aron et al., 2003). Strikingly, activation of the right IFG was related to the speed of the inhibition process and use of illegal substances in a large sample of adolescents (Whelan et al., 2012), and a single administration of testosterone altered the connectivity between the IFG and the ACC in healthy females (Bos et al., 2016). Although not representing a specific involvement (Wager et al., 2016), the critical role of the dorsal ACC in cognitive, motor and attentional control is well established (Bush et al., 2000), and along with implications of the dorsal ACC for reward-based decision-making (Bush et al., 2002), reduced connectivity with the DAN may reflect impaired coordinated neuronal processing related to cognitive control and impulsivity in AAS users. Our current findings are in line with a previous magnetic resonance spectroscopy (MRS) study reporting neurochemical abnormalities in the dorsal ACC in AAS users, likely reflecting increased glutamate turnover (Kaufman et al., 2015).

In contrast to the DMN-amygdala connection, the SFG/IFG/ACC-DAN edge was lower also in previous users compared to controls, and further reductions with increased dependency and lifetime exposure suggest an association with sustained use. This fits with the observation of reduced cognitive function after long-term use. Although further studies are needed to establish the reliability and state-dependency of these findings, they may suggest a chronic state conferring risk of long-term use and cognitive impairment.

In line with the state-dependency of functional imaging indices, the effects seems to be partly driven by current use, underscored by correlations between connectivity in both edges and T/E ratio, and the lack of differences between non-users and previous users on the amygdala-DMN edge. This could reflect the transient and state-dependent nature of resting state patterns, partly mediated by hormonal fluctuations. Typical AAS administration regiments results in highly non-physiological levels of androgens and their metabolites, and large fluctuations at different time points in the cycle. Hormonal fluctuations of this kind are likely to influence brain functions, and might explain AAS induced alterations in mood and behavior (Pope and Katz, 1994, Su et al., 1993).

This study does not come without limitation. First, the study design does not allow for claims regarding causality. In line with most observational and correlational studies, it is conceivable that predisposed variables conferring risk for AAS use (personality etc) may reflect confounding factors. Effects of such inherent associations should ideally be handled by design, e.g. by a prospective and randomized experimental study. Further, whereas the results suggest the involvement of selective large-scale brain functional networks, the neurobiological specificity of fMRI-based data is limited, which complicates a direct mechanistic interpretation. Indeed, the actions of AAS are complex and multifactorial, including both the described genomic effects but also additional non-genomic actions, estrogen receptor binding, and secondary modulatory effects on the hypothalamic-pituitary-adrenal (HPA) axis and influence of classical neurotransmitter and neuropeptide signaling. Further, the diversity of AAS effects on behavior reflects interactions between many factors, including predisposing vulnerability, age, sex, type(s) of AAS used, doses and method of administration (Oberlander and Henderson, 2012). Although the observed correlations with T/E ratio suggest an acute effect of AAS, the associations with dependency and life time exposure also indicate a cumulative effect of sustained use. Further studies are needed to disentangle the acute and chronic influences of AAS use on brain and cognitive function, which is critical for informing predictive and prognostic models of the long-term impact of AAS use. In line with the reported sex differences in AAS use (Kanayama et al., 2007, Pope et al., 2014), we included male subjects only. Further studies are needed to address the generalizability to the female population. Lastly, the consequences of AAS use are not confined to the brain. In particular, cardiovascular conditions, including hypertension, atherosclerosis and dyslipidemia, are substantial risks associated with AAS use (Vanberg and Atar, 2010). These cardiovascular risk factors could also influence the fMRI signal, which is modulated by complex neurovascular interactions (Logothetis, 2008), and future studies may be able to directly assess the common and unique effects on the brain and the cardiovascular system.

In conclusion, in the largest sample to date we have demonstrated reduced resting-state functional connectivity between brain network nodes critically involved in cognitive and emotional regulation, including the amygdala, the DMN, the DAN and SFG/IFG/ACC in AAS users. The brain connectivity findings are in line with the characteristic neuropsychiatric and cognitive consequences of sustained AAS use, including emotional and behavioral dysregulation. Associations with T/E ratio support sensitivity to current hormonal levels, and connectivity reductions with increased life time exposure and dependency suggest cumulative effects of sustained AAS use.

## Author contributions

LTW, TK and AB wrote the manuscript; LTW, DA, TK, IU and AB analyzed data, AB collected the data. All authors reviewed and approved the manuscript.

## Financial disclosures

All authors report no biomedical financial interests or potential conflicts of interest.

## Figures and Tables

**Table 1:** Group comparisons on main attributes; demographic information, training information, drug and psychopharmaca use, emotional and behavioral problems.

	Controls (n = 59)		Current AAS users (n = 50)		Previous AAS users (n = 16)				
	M	SD	M	SD	M	SD	F	p	
Age (years)	30.7	7.4	33.6	8.7	31.7	5.2	1.967	0.144	
Education (years)	15.9	2.7	14.5	2.6	14.2	1.9	5.230	0.007	a⁎
IQ	112.7	9.5	105.9	12.6	107.3	10.1	5.518	0.005	a⁎⁎
Height	180.7	6.5	181.1	6.9	180.5	6.9	0.084	0.920	
Weight	90.2	14.5	99.1	12.0	93.9	15.4	5.737	0.004	a⁎⁎
BMI	27.6	4.0	30.2	3.6	28.7	4.0	6.243	0.003	a⁎⁎
Strength training/week (min)	477.3	247.4	383.7	217.4	223.3	123.2	8.600	< 0.001	b⁎⁎⁎, c⁎
Endurance training/week (min)	92.9	118.4	115.8	197.1	78.1	106.1	0.481	0.619	
Cigarettes (day)	0.4	2.6	1.8	4.3	0.6	2.3	2.631	0.076	
Alcohol units (week)	3.5	5.1	1.6	3.2	1.4	2.3	3.600	0.030	a⁎
Psychopharmaca (previous or current use)	n	%	n	%	n	%			
Antidepressants	2	3.4	10	20.0	3	18.8			
Anxiolytics	0	0.0	7	14.0	2	12.5			
Opioids	0	0.0	2	4.0	0	0.0			
More than one sort	0	0.0	3	6.0	0	0.0			
None reported	55	93.2	37	74.0	11	68.8			
Emotional and problem behavior									
Anxious/depressed	52.2	4.3	54.5	6.7	58.6	9.0	6.300	0.003	b⁎⁎
Rule breaking behavior	52.5	5.1	59.3	11.0	58.9	7.9	9.197	< 0.001	a⁎⁎⁎, b⁎
Internalizing problems	45.9	9.9	51.3	11.0	56.3	13.3	5.958	0.004	a⁎, b⁎⁎
Externalizing problems	46.4	8.4	55.0	8.8	55.7	11.1	13.172	< 0.001	a⁎⁎⁎,b⁎⁎
Total problems	21.1	15.6	33.8	21.2	42.8	25.5	8.752	< 0.001	a⁎⁎, b⁎⁎
Tobacco	55.9	5.3	54.1	4.9	55.8	5.6	1.501	0.228	
Alcohol	60.1	7.0	56.8	6.8	59.5	8.5	2.637	0.076	
Drugs	51.4	7.0	57.5	12.9	56.6	9.8	4.564	0.013	a⁎⁎

Bonferroni Post Hoc test a = control significantly different from current users, b = controls significantly different from previous users, c = current AAS users significantly different from previous AAS users.

⁎ p < 0.05.

⁎⁎ p < 0.01.

⁎⁎⁎ p < 0.001.

## Appendix A Supplementary data

Supplementary material Image 1

## References

[bb0005] Alnaes D., Kaufmann T., Richard G., Duff E.P., Sneve M.H., Endestad T., Nordvik J.E., Andreassen O.A., Smith S.M., Westlye L.T. (2015). Attentional load modulates large-scale functional brain connectivity beyond the core attention networks. NeuroImage.

[bb0010] Andersson J.L.R., Jenkinson M., Smith S.M. (2010). Non-linear Registration, AKA Spatial Normalisation. FMRIB Technical Report TR07JA2.

[bb0015] Aron A.R., Fletcher P.C., Bullmore E.T., Sahakian B.J., Robbins T.W. (2003). Stop-signal inhibition disrupted by damage to right inferior frontal gyrus in humans. Nat. Neurosci..

[bb0020] Aron A.R., Robbins T.W., Poldrack R.A. (2014). Inhibition and the right inferior frontal cortex: one decade on. Trends Cogn. Sci..

[bb0025] Basile J.R., Binmadi N.O., Zhou H., Yang Y.H., Paoli A., Proia P. (2013). Supraphysiological doses of performance enhancing anabolic-androgenic steroids exert direct toxic effects on neuron-like cells. Front. Cell. Neurosci..

[bb0030] Beckmann C.F., Smith S.M. (2004). Probabilistic independent component analysis for functional magnetic resonance imaging. IEEE Trans. Med. Imaging.

[bb0035] Bjørnebekk A., Walhovd K.B., Jørstad M., Due-Tonnessen P., Hullstein I., Fjell A.M. (2016). Structural brain imaging of long term anabolic-androgenic steroid users and non-using weightlifters. Biol. Psychiatry.

[bb0040] Bos P.A., Hofman D., Hermans E.J., Montoya E.R., Baron-Cohen S., van Honk J. (2016). Testosterone reduces functional connectivity during the ‘Reading the Mind in the Eyes’ test. Psychoneuroendocrinology.

[bb0045] Buckner R.L., Andrews-Hanna J.R., Schacter D.L. (2008). The brain's default network: anatomy, function, and relevance to disease. Ann. N. Y. Acad. Sci..

[bb0050] Bush G., Luu P., Posner M.I. (2000). Cognitive and emotional influences in anterior cingulate cortex. Trends Cogn. Sci..

[bb0055] Bush G., Vogt B.A., Holmes J., Dale A.M., Greve D., Jenike M.A., Rosen B.R. (2002). Dorsal anterior cingulate cortex: a role in reward-based decision making. Proc. Natl. Acad. Sci. U. S. A..

[bb0060] Caraci F., Pistara V., Corsaro A., Tomasello F., Giuffrida M.L., Sortino M.A., Nicoletti F., Copani A. (2011). Neurotoxic properties of the anabolic androgenic steroids nandrolone and methandrostenolone in primary neuronal cultures. J. Neurosci. Res..

[bb0065] Clark A.S., Mitre M.C., Brinck-Johnsen T. (1995). Anabolic-androgenic steroid and adrenal steroid effects on hippocampal plasticity. Brain Res..

[bb0070] Corbetta M., Patel G., Shulman G.L. (2008). The reorienting system of the human brain: from environment to theory of mind. Neuron.

[bb0075] Cunningham R.L., Giuffrida A., Roberts J.L. (2009). Androgens induce dopaminergic neurotoxicity via caspase-3-dependent activation of protein kinase Cdelta. Endocrinology.

[bb0080] du Boisgueheneuc F., Levy R., Volle E., Seassau M., Duffau H., Kinkingnehun S., Samson Y., Zhang S., Dubois B. (2006). Functions of the left superior frontal gyrus in humans: a lesion study. Brain.

[bb0085] Estrada M., Varshney A., Ehrlich B.E. (2006). Elevated testosterone induces apoptosis in neuronal cells. J. Biol. Chem..

[bb0090] Filippini N., MacIntosh B.J., Hough M.G., Goodwin G.M., Frisoni G.B., Smith S.M., Matthews P.M., Beckmann C.F., Mackay C.E. (2009). Distinct patterns of brain activity in young carriers of the APOE-epsilon4 allele. PNAS.

[bb0095] Fischl B., Salat D.H., Busa E., Albert M., Dieterich M., Haselgrove C., van der Kouwe A., Killiany R., Kennedy D., Klaveness S., Montillo A., Makris N., Rosen B., Dale A.M. (2002). Whole brain segmentation: automated labeling of neuroanatomical structures in the human brain. Neuron.

[bb0100] Fuster J.M. (2001). The prefrontal cortex–an update: time is of the essence. Neuron.

[bb0105] Greve D.N., Fischl B. (2009). Accurate and robust brain image alignment using boundary-based registration. NeuroImage.

[bb0110] group (2016). WADA Technical Document - TD2016EAAS. https://www.wada-ama.org/en/resources/science-medicine/td2016-eaas.

[bb0115] Hall R.C., Chapman M.J. (2005). Psychiatric complications of anabolic steroid abuse. Psychosomatics.

[bb0120] Heany S.J., van Honk J., Stein D.J., Brooks S.J. (2016). A quantitative and qualitative review of the effects of testosterone on the function and structure of the human social-emotional brain. Metab. Brain Dis..

[bb0125] Heffernan T., Battersby L., Bishop T., O'Neill T. (2015). The everyday cognitive consequences of regular use of anabolic androgenic steroids in a sporting context. Eur. Psychiatry.

[bb0130] Heffernan T.M., Battersby L., Bishop P., O'Neill T.S. (2015). Everyday memory deficits associated with anabolic-androgenic steroid use in regular gymnasium users. Open Psychiatry J..

[bb0135] Heinlein C.A., Chang C. (2002). The roles of androgen receptors and androgen-binding proteins in nongenomic androgen actions. Mol. Endocrinol..

[bb0140] Hullstein I.R., Malerod-Fjeld H., Dehnes Y., Hemmersbach P. (2015). Black market products confiscated in Norway 2011–2014 compared to analytical findings in urine samples. Drug Test. Anal..

[bb0145] Ip E.J., Barnett M.J., Tenerowicz M.J., Perry P.J. (2011). The Anabolic 500 survey: characteristics of male users versus nonusers of anabolic-androgenic steroids for strength training. Pharmacotherapy.

[bb0150] Janne O.A., Palvimo J.J., Kallio P., Mehto M. (1993). Androgen receptor and mechanism of androgen action. Ann. Med..

[bb0155] Jenkinson M., Smith S. (2001). A global optimisation method for robust affine registration of brain images. Med. Image Anal..

[bb0160] Kanayama G., Pope H.G., Hudson J.I. (2001). “Body image” drugs: a growing psychosomatic problem. Psychother. Psychosom..

[bb0165] Kanayama G., Boynes M., Hudson J.I., Field A.E., Pope H.G. (2007). Anabolic steroid abuse among teenage girls: an illusory problem?. Drug Alcohol Depend..

[bb0170] Kanayama G., Brower K.J., Wood R.I., Hudson J.I., Pope H.G. (2009). Anabolic-androgenic steroid dependence: an emerging disorder. Addiction.

[bb0175] Kanayama G., Hudson J.I., Pope H.G. (2009). Features of men with anabolic-androgenic steroid dependence: a comparison with nondependent AAS users and with AAS nonusers. Drug Alcohol Depend..

[bb0180] Kanayama G., Kean J., Hudson J.I., Pope H.G. (2012). Cognitive deficits in long-term anabolic-androgenic steroid users. Drug Alcohol Depend..

[bb0185] Kaufman M.J., Janes A.C., Hudson J.I., Brennan B.P., Kanayama G., Kerrigan A.R., Jensen J.E., Pope H.G. (2015). Brain and cognition abnormalities in long-term anabolic-androgenic steroid users. Drug Alcohol Depend..

[bb0190] Kaufmann T., Skatun K.C., Alnaes D., Doan N.T., Duff E.P., Tonnesen S., Roussos E., Ueland T., Aminoff S.R., Lagerberg T.V., Agartz I., Melle I.S., Smith S.M., Andreassen O.A., Westlye L.T. (2015). Disintegration of sensorimotor brain networks in schizophrenia. Schizophr. Bull..

[bb0195] Kaufmann T., Elvsashagen T., Alnaes D., Zak N., Pedersen P.O., Norbom L.B., Quraishi S.H., Tagliazucchi E., Laufs H., Bjornerud A., Malt U.F., Andreassen O.A., Roussos E., Duff E.P., Smith S.M., Groote I.R., Westlye L.T. (2016). The brain functional connectome is robustly altered by lack of sleep. NeuroImage.

[bb0200] Keller E.T., Ershler W.B., Chang C. (1996). The androgen receptor: a mediator of diverse responses. Front. Biosci..

[bb0205] Kelly R.E., Alexopoulos G.S., Wang Z., Gunning F.M., Murphy C.F., Morimoto S.S., Kanellopoulos D., Jia Z., Lim K.O., Hoptman M.J. (2010). Visual inspection of independent components: defining a procedure for artifact removal from fMRI data. J. Neurosci. Methods.

[bb0210] Kritzer M. (2004). The distribution of immunoreactivity for intracellular androgen receptors in the cerebral cortex of hormonally intact adult male and female rats: localization in pyramidal neurons making corticocortical connections. Cereb. Cortex.

[bb0215] Ledoit O., Wolf M. (2003). Improved estimation of the covariance matrix of stock return with an application to portfolio selection. J. Empir. Financ..

[bb0220] Li W., Qin W., Liu H., Fan L., Wang J., Jiang T., Yu C. (2013). Subregions of the human superior frontal gyrus and their connections. NeuroImage.

[bb0225] Li W., Mai X., Liu C. (2014). The default mode network and social understanding of others: what do brain connectivity studies tell us. Front. Hum. Neurosci..

[bb0230] Logothetis N.K. (2008). What we can do and what we cannot do with fMRI. Nature.

[bb0235] Mareck U., Geyer H., Fussholler G., Schwenke A., Haenelt N., Piper T., Thevis M., Schanzer W. (2010). Reporting and managing elevated testosterone/epitestosterone ratios–novel aspects after five years' experience. Drug Test. Anal..

[bb0240] Menard C.S., Harlan R.E. (1993). Up-regulation of androgen receptor immunoreactivity in the rat brain by androgenic-anabolic steroids. Brain Res..

[bb0245] Michael R.P., Clancy A.N., Zumpe D. (1995). Distribution of androgen receptor-like immunoreactivity in the brains of cynomolgus monkeys. J. Neuroendocrinol..

[bb0250] Oberlander J.G., Henderson L.P. (2012). The Sturm und Drang of anabolic steroid use: angst, anxiety, and aggression. Trends Neurosci..

[bb0255] Orlando R., Caruso A., Molinaro G., Motolese M., Matrisciano F., Togna G., Melchiorri D., Nicoletti F., Bruno V. (2007). Nanomolar concentrations of anabolic-androgenic steroids amplify excitotoxic neuronal death in mixed mouse cortical cultures. Brain Res..

[bb0260] Pagonis T.A., Angelopoulos N.V., Koukoulis G.N., Hadjichristodoulou C.S. (2006). Psychiatric side effects induced by supraphysiological doses of combinations of anabolic steroids correlate to the severity of abuse. Eur. Psychiatry.

[bb0265] Pagonis T.A., Angelopoulos N.V., Koukoulis G.N., Hadjichristodoulou C.S., Toli P.N. (2006). Psychiatric and hostility factors related to use of anabolic steroids in monozygotic twins. Eur. Psychiatry.

[bb0270] Peters S., Jolles D.J., Van Duijvenvoorde A.C., Crone E.A., Peper J.S. (2015). The link between testosterone and amygdala-orbitofrontal cortex connectivity in adolescent alcohol use. Psychoneuroendocrinology.

[bb0275] Phelps E.A., LeDoux J.E. (2005). Contributions of the amygdala to emotion processing: from animal models to human behavior. Neuron.

[bb0280] Pomerantz S.M., Fox T.O., Sholl S.A., Vito C.C., Goy R.W. (1985). Androgen and estrogen receptors in fetal rhesus monkey brain and anterior pituitary. Endocrinology.

[bb0285] Pope H.G., Katz D.L. (1994). Psychiatric and medical effects of anabolic-androgenic steroid use. A controlled study of 160 athletes. Arch. Gen. Psychiatry.

[bb0290] Pope H.G., Kouri E.M., Hudson J.I. (2000). Effects of supraphysiologic doses of testosterone on mood and aggression in normal men: a randomized controlled trial. Arch. Gen. Psychiatry.

[bb0295] Pope H.G., Kanayama G., Athey A., Ryan E., Hudson J.I., Baggish A. (2014). The lifetime prevalence of anabolic-androgenic steroid use and dependence in Americans: current best estimates. Am. J. Addict..

[bb0300] Pruim R.H., Mennes M., Buitelaar J.K., Beckmann C.F. (2015). Evaluation of ICA-AROMA and alternative strategies for motion artifact removal in resting state fMRI. NeuroImage.

[bb0305] Reyes-Fuentes A., Veldhuis J.D. (1993). Neuroendocrine physiology of the normal male gonadal axis. Endocrinol. Metab. Clin. N. Am..

[bb0310] Roalf D.R., Quarmley M., Elliott M.A., Satterthwaite T.D., Vandekar S.N., Ruparel K., Gennatas E.D., Calkins M.E., Moore T.M., Hopson R., Prabhakaran K., Jackson C.T., Verma R., Hakonarson H., Gur R.C., Gur R.E. (2016). The impact of quality assurance assessment on diffusion tensor imaging outcomes in a large-scale population-based cohort. NeuroImage.

[bb0315] Salimi-Khorshidi G., Douaud G., Beckmann C.F., Glasser M.F., Griffanti L., Smith S.M. (2014). Automatic denoising of functional MRI data: combining independent component analysis and hierarchical fusion of classifiers. NeuroImage.

[bb0320] Simerly R.B., Chang C., Muramatsu M., Swanson L.W. (1990). Distribution of androgen and estrogen receptor mRNA-containing cells in the rat brain: an in situ hybridization study. J. Comp. Neurol..

[bb0325] Skatun, K.C., Kaufmann, T., Doan, N.T., Alnaes, D., Cordova-Palomera, A., Jonsson, E.G., Fatouros-Bergman, H., Flyckt, L., KASP, Melle, I., Andreassen, O.A., Agartz, I., Westlye, L.T. Consistent functional connectivity aberrations in schizophrenia spectrum disorder: a multi-site study. Schizophr. Bull. (in press), http://dx.doi.org/10.1093/schbul/sbw145. 10.1093/schbul/sbw145 PMC5515107 27872268

[bb0330] Smith S.M., Jenkinson M., Woolrich M.W., Beckmann C.F., Behrens T.E., Johansen-Berg H., Bannister P.R., De Luca M., Drobnjak I., Flitney D.E., Niazy R.K., Saunders J., Vickers J., Zhang Y., De Stefano N., Brady J.M., Matthews P.M. (2004). Advances in functional and structural MR image analysis and implementation as FSL. NeuroImage.

[bb0335] Smith S.M., Miller K.L., Salimi-Khorshidi G., Webster M., Beckmann C.F., Nichols T.E., Ramsey J.D., Woolrich M.W. (2011). Network modelling methods for FMRI. NeuroImage.

[bb0340] Smith S.M., Hyvarinen A., Varoquaux G., Miller K.L., Beckmann C.F. (2014). Group-PCA for very large fMRI datasets. NeuroImage.

[bb0345] Spielberg J.M., Forbes E.E., Ladouceur C.D., Worthman C.M., Olino T.M., Ryan N.D., Dahl R.E. (2015). Pubertal testosterone influences threat-related amygdala-orbitofrontal cortex coupling. Soc. Cogn. Affect. Neurosci..

[bb0350] Su T.P., Pagliaro M., Schmidt P.J., Pickar D., Wolkowitz O., Rubinow D.R. (1993). Neuropsychiatric effects of anabolic steroids in male normal volunteers. JAMA.

[bb0355] Thiblin I., Petersson A. (2005). Pharmacoepidemiology of anabolic androgenic steroids: a review. Fundam. Clin. Pharmacol..

[bb0360] Trenton A.J., Currier G.W. (2005). Behavioural manifestations of anabolic steroid use. CNS Drugs.

[bb0365] van Wingen G.A., Ossewaarde L., Backstrom T., Hermans E.J., Fernandez G. (2011). Gonadal hormone regulation of the emotion circuitry in humans. Neuroscience.

[bb0370] Vanberg P., Atar D. (2010). Androgenic anabolic steroid abuse and the cardiovascular system. Handb. Exp. Pharmacol..

[bb0375] Volman I., Toni I., Verhagen L., Roelofs K. (2011). Endogenous testosterone modulates prefrontal-amygdala connectivity during social emotional behavior. Cereb. Cortex.

[bb0380] Wager T.D., Atlas L.Y., Botvinick M.M., Chang L.J., Coghill R.C., Davis K.D., Iannetti G.D., Poldrack R.A., Shackman A.J., Yarkoni T. (2016). Pain in the ACC?. Proc. Natl. Acad. Sci. U. S. A..

[bb0385] Whelan R., Conrod P.J., Poline J.B., Lourdusamy A., Banaschewski T., Barker G.J., Bellgrove M.A., Buchel C., Byrne M., Cummins T.D., Fauth-Buhler M., Flor H., Gallinat J., Heinz A., Ittermann B., Mann K., Martinot J.L., Lalor E.C., Lathrop M., Loth E., Nees F., Paus T., Rietschel M., Smolka M.N., Spanagel R., Stephens D.N., Struve M., Thyreau B., Vollstaedt-Klein S., Robbins T.W., Schumann G., Garavan H., Consortium I. (2012). Adolescent impulsivity phenotypes characterized by distinct brain networks. Nat. Neurosci..

[bb0390] Whitfield-Gabrieli S., Ford J.M. (2012). Default mode network activity and connectivity in psychopathology. Annu. Rev. Clin. Psychol..

