# Supplementary material for: Brain connectivity aberrations in anabolic-androgenic steroid users
Source: Neuroimage Clin. 2016 Nov 17;13:62–9. doi: 10.1016/j.nicl.2016.11.014 (PMC5133655; doi:10.1016/j.nicl.2016.11.014)
Supplement: Supplementary file 1 — Supplementary material [file mmc1.doc]

**Supplemental Material**

**Methods**

*Semi-structured interview*

A semi-structured interview was designed for the purpose of the study and administered at the same site visit as the neuropsychological evaluation. The interview consisted of questions related to age and education, and to health and exercise habits, i.e. number of weightlifting vs. endurance training sessions per week, training identity, their personal lift records in classic powerlifting exercises, and any achievements in sports they have participated in. They were asked about smoking habits and alcohol consumption, disease history including previous head trauma, use of medication and psychopharmaca and substance abuse. AAS users were also interviewed about the nature of their AAS use, such as motives behind their usage, age of onset, administration pattern, length of cycles and number of life-time cycles, average weekly dosage, where in the cycle they were presently (at the time of assessment), whether and when they have ceased using AAS. Any medical/physical, emotional/psychological and cognitive side effects they had noticed were recorded. The participants in the AAS group also completed a form mapping which AAS substances, and other doping agents they have ever used, and asked to rank the top five used substances in order of usage amount and enter the commonly used dosage for these.

*AAS lifetime dose estimate*

There are many factors that might influence the effect of AAS, e.g. different properties of the compounds used, and the use of illegally “basement-produced” steroids. In addition self-reports of doses used, and periods of use in the past are not necessarily very accurate. Thus estimates of the quantity of AAS used over a lifetime that are based upon calculations of various compounds and the periods of use, are likely not very precise . Hence, considering these limitations we here applied a grouping index categorizing accumulated lifetime AAS doses (i.e., lifetime weeks of AAS and testosterone use multiplied by average weekly doses used) into low, medium and high (based upon splitting all study participants into three equal sized groups). Accumulated lifetime doses below 126 000 mg were classified as *low*, between 126001-368000 mg as *medium*, and accumulated lifetime use exceeding 368001 mg as *high*.

*AAS subgroups*

Various subsamples were used in order to better understand the relationships between AAS use and resting state functional connectivity. Below we have described the criteria we have used in order to define such subcategories of AAS users and controls.

AAS-testosterone

Since the potential influence of AAS use on resting state connectivity is the main focus of the paper, the main analysis constitute a comparison of current AAS users testing positive for AAS versus non-users whose drug tests was negative. However this subdivision has its weaknesses. First, it does not take into account participants who only administer testosterone compounds. Second, it does not take into account the various detection times of the compounds used, thus does not always separate AAS users from controls. Since exogenous testosterone administration will be reflected in the testosterone to epitestosterone (T/E) ratio, an additional classifier used to determine the use of exogenous testosterone was T/E ratios above 4, as has been applied by World Anti-Doping Agency (WADA) (32). Hence, the AAS-testosterone group comprises current users (see below) with a self-reported history of AAS use and with urine test indicative of AAS or testosterone use defined by these criteria. The control group includes participants with no AAS history and a T/E ratio below 4 (4 participants with T/E ratio >4 were excluded).

The urinary T/E ratio consists of testosterone glucuronide and epitestosterone glucuronide in addition to the free form of T and E (which are normally < 5%). In a European population the ratio will show a gaussian distribution and the peak will be around 1. Due to genetic variation (in the gene responsible for the glucuronidation) the values show a considerable interindividual variation. Some individuals may have a naturally elevated T/E ratio, but T/E >10 are very rare in absence of testosterone administration. A person that has naturally elevated T/E does not necessarily have higher testosterone in serum. It is therefore necessary to interpret the T/E ratio with care. Several factors may influence the ratio, including ethanol consumption (short time effect after large amount).

Previous or current AAS use

As the half-lives of different AAS compounds vary widely it is not obvious where a clear boundary between being a current and previous user should be put. Moreover, some users are only taking one AAS cycle a year and could have periods of several months without AAS. In order to clearly separate current users off-cycle from previous users, and with the idea that the brain tissue also should be given some time to potentially recuperate, we chose to apply a one-year boundary since last usage in order to classify as a previous AAS user.

AAS dependence

The presence of AAS dependence was evaluated by a structured diagnostic interview module in the format of the SCID II . The interview is based upon the standard substance-dependence criteria of DSM-IV, but modified and adapted to apply to AAS dependence . It has shown promising psychometric properties in a preliminary study by Pope, Kean and colleagues .

*Potential confounders*

IQ

The vocabulary and the matrix reasoning subtests from the Wechslers Abbreviated Scale of Intelligence (PsychCorp, 1999) were applied to get an estimate of general intelligence. The WASI Vocabulary test is a 42-item task similar to the Vocabulary subtests of the WISC-III and the WAIS-III. Vocabulary is a measure of the individual’s expressive vocabulary, verbal knowledge, and fund of information. It is thought to be a good measure of crystallized and general intelligence, or g . Matrix reasoning consists of 35 incomplete grid patterns that require the participant to select a correct response from five possible choices.

Weekly alcohol consumption

Self- reported weekly alcohol consumption (in standard units) was recorded, and used to test and control for mediating effects of alcohol use on the relationships between group belonging and imaging parameters.

ASEBA ASR scales

In order to control for recent use of illegal drugs, mood symptoms and problem behaviour we used selected scales from the ASEBA Adult Self Report (ASR). The ASR is a revision of the Youth Adult Self Report protocol for adults aged 18-59 , originally derived from the widely used Child Behaviour Checklist (CBCL). It was designed to assess emotional and behavioural problems in a standardized format. The ASR contains 126 items on behaviour that has occurred over the past 6 months. The items are scored on a three-point scale: 0 (not true), 1 (somewhat or sometimes true), and 2 (very true or often true). Good reliability and validity have been shown in American and Dutch samples (26). The scales applied are *Anxious/Depressed* capturing symptoms of both anxiety and depression (i.e. fearful, cries a lot, worries), the *Drug-scale* providing a measure of use of illegal drugs during the past 6 months, *Attention problems* (i.e. cannot concentrate, forgetful, disorganized), and the *Total Problems Score* that is a scale summarizing the individual item scores thus providing global indices of the individual’s competencies and problems.

*Comparison between current, previous and excluded users*

Supplemental Table 1 shows summary stats per group and comparisons between current, previous and excluded users on key use characteristics. No significant differences were found.

|  | **Current users**  n=50 | | | **Previous**  **users**  n=16 | | | **Excluded**  **users**  n=15 | | | |  | |  |
| --- | --- | --- | --- | --- | --- | --- | --- | --- | --- | --- | --- | --- | --- |
|  |  | |  |
| **Attribute** | Mean or *n* | SD or % | Mean or *n* (%) | | SD or % | Mean or *n* (%) | | SD or % | **F** | ***P*** | |  | |
| Debut age | 22.6 | 5.7 | 20.0 | | 3.6 | 21.8 | | 9.4 | 1.02 | 0.36 | |  | |
| Years of AAS use | 9.4 | 5.9 | 7.7 | | 3.6 | 10.2 | | 5.8 | 0.87 | 0.42 | |  | |
| Average weekly AAS dose,  mg | 1322 | 1205 | 1325 | | 754 | 1165 | | 933 | 0.12 | 0.89 | |  | |
| Cumulative lifetime AAS  dose, mg | 411786 | 627270 | 385377 | | 389047 | 245089 | | 244504 | 0.52 | 0.60 | |  | |
| AAS dependency | 26 | 53.1 | 10 | | 62.5 | 7 | | 46.7 |  |  | |  | |
|  |  |  |  | |  |  | |  |  |  | |  | |

**Supplemental Table 1.** AAS use characteristics. Summary statistics and group comparisons on key use characteristics between current users with positive tests, previous users with negative tests and AAS users who were excluded from group analyses due to negative doping test. No significant differences between groups were found.


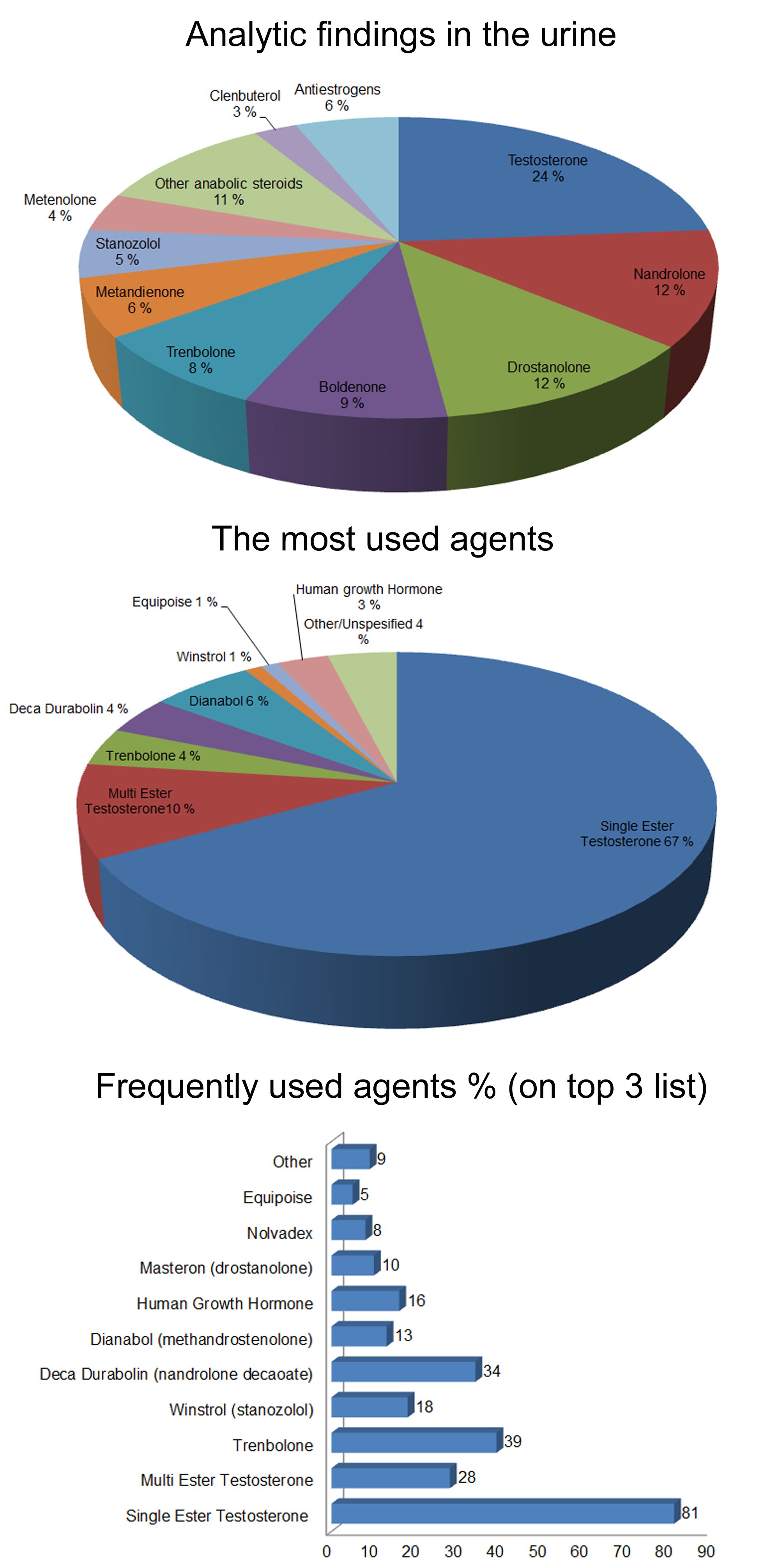


**Supplemental Figure 1.** The distribution of the various anabolic-androgenic steroids found in the urine sample, based upon a total of 194 positive findings. A testosterone/epitestosterone ratio > 4 was used as criterion for testosterone in the samples collected (upper row). The doping analyses findings are based upon data from the full sample of 142 males. 94% of the positive findings stemmed from current users, whereas five tests with T/E ratio > 4 where detected in the control group (excluded from group analyses). Further in the previous user group 4 were excluded from group comparisons due to positive tests of testosterone (n=2), traces of AASs (n=1) or antiestrogens (n=1). Self-reports show that single ester testosterones (mainly testosterone enanthate) are the most popular doping substances, listed as the mostly used agent by 67 % of the previous or current AAS users (middle row). When asked to list the three most used agents 81 % listed single ester testosterone agents (including testosterone enanthate, proprionate and unspecified testosterone)(bottom row). Note that 22% of the AAS-group included two single ester testosterone agents among their top three used substances (not shown). Data were available for 79 previous or current AAS users.

|  | Whole sample  n=142 | |  | Current users  n=50 | |
| --- | --- | --- | --- | --- | --- |
|  | *n* | (%) |  | *n* | (%) |
| Testosterone | 46 | 24 |  | 37 | 74 |
| Nandrolone (Deca Durabolin) | 24 | 12 |  | 24 | 48 |
| Drostanolone (Masteron) | 23 | 12 |  | 20 | 40 |
| Boldenone (Equipose) | 17 | 9 |  | 16 | 32 |
| Trenbolone | 16 | 8 |  | 16 | 32 |
| Metandienone (Dianabol) | 12 | 6 |  | 12 | 24 |
| Stanozolol (Winstrol) | 10 | 5 |  | 8 | 16 |
| Metenolone (Primobolan) | 8 | 4 |  | 7 | 14 |
| Other anabolic steroids | 21 | 11 |  | 7 | 14 |
| Clenbuterol | 5 | 3 |  | 3 | 6 |
| Antiestrogens | 12 | 6 |  | 14 | 28 |
| Sum positive tests | 194 |  |  | 164 |  |

**Supplemental Table 2.** The number and distribution of the analytic findings of various anabolic-androgenic steroids and other doping agents found in the urine samples. A testosterone/epitestosterone ratio > 4 was used as criterion for testosterone in the samples collected. The full sample of 142 males contained 194 positive findings. 94% of the positive findings stemmed from current users. The percentages for the full sample show the relative distribution of the identified agents, whereas the percentages for the current users show the proportion of current users with positive tests for each agent.

|  | Most frequently used agent (1st ranked) | | Frequently used agents (top 3 ranked) | |
| --- | --- | --- | --- | --- |
|  | *n* | % | *n* | % |
| Testosterone (single ester) | 53 | 67 | 64 | 81 |
| Testosterone (multi ester) | 8 | 10 | 22 | 28 |
| Trenbolone | 3 | 4 | 31 | 39 |
| Deca Durabolin (nandrolone decanoate) | 3 | 4 | 27 | 34 |
| Dianabol (metandienone, methandrostenolone) | 5 | 6 | 10 | 13 |
| Winstrol (stanozolol) | 1 | 1 | 14 | 18 |
| Human growth Hormone | 2 | 3 | 13 | 16 |
| Equipoise (boldenone undecanoate) | 1 | 1 | 4 | 5 |
| Masteron (drostanolone) |  |  | 8 | 10 |

**Supplemental Table 3.** Single ester testosterones (mainly testosterone enanthate) are the most popular doping substances, ranked as the mostly used agent by 67 % of the previous or current AAS users (middle row). When asked to rank the three most used agents 81 % listed single ester testosterone agents (including testosterone enanthate, propionate and unspecified testosterone). Note that 22% of the AAS-group included two single ester testosterone agents among their top three used substances (not shown). Data were available for 79 previous or current AAS users.

*fMRI processing and analysis*

We did not regress out the global signal, as global signal regression decreases signal-to-noise ratio (SNR) . Rather, we used regularized partial correlation matrices to infer connectivity, known to be relatively unaffected by global signal .

Effects of FIX data cleaning on tSNR

Supplemental Figure 1 shows average temporal signal-to-noise ratio (tSNR) before and after FIX. FIX dramatically increased tSNR in all subjects (paired samples t-test: t=52.93, p=5e-87; average increase: 37.86%, SD: 14.09%, range: 19.3-126.5%). After FIX, current AAS users showed lower tSNR compared to controls (t=3.93, p=.00015) yet there were no significant differences between previous and non-users (t=0.03, p=.98) or between current and previous users (t=2.3, p=.02).


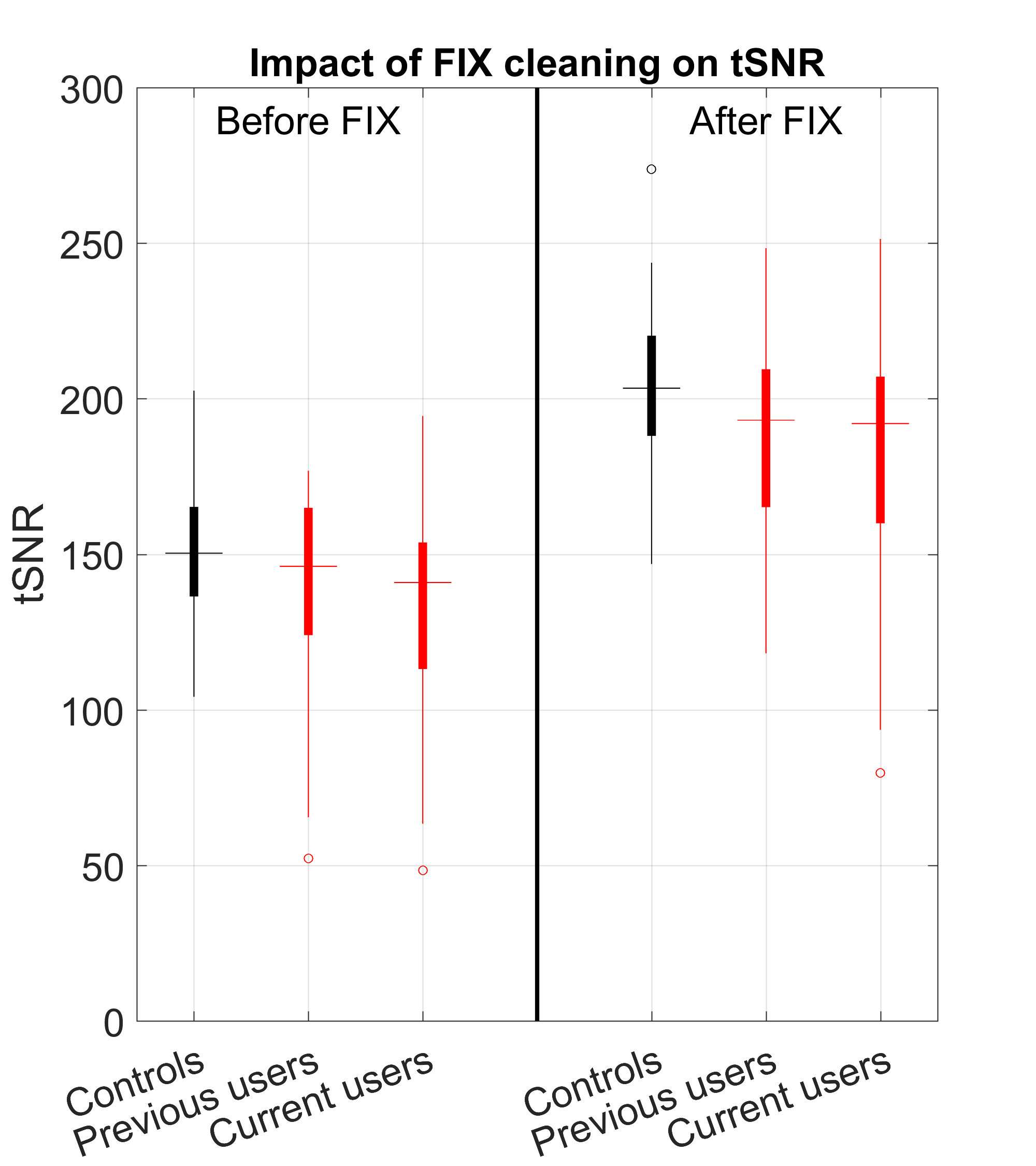


**Supplemental Figure 2.** tSNR before and after FIX in each of the three groups.

**Results**

*Drug analyses*

The urine toxicology findings of narcotics were in accordance with the participants’ self-reports of the drugs popularity recently presented . Briefly, among the 125 individuals included in the main analysis, traces of narcotics were found in 22. Traces after the use of marijuana (THC) and cocaine were the most frequent, detected in 9 (7.2%) and 8 (6.4%) samples, respectively. Out of these, 6 THC and 5 cocaine findings stemmed from current AAS users, 2 of each drug from non-users, and 1 of each drug from previous users. No other illegal drugs were found in the control group (besides 3 cases of codeine/morphine resembling the use of prescribed cough medicine and painkillers). Furthermore, 4 tests were positive for amphetamines, and 5 (partly overlapping) for traces of Ritalin likely comprising both illegal and prescribed variants, all in current AAS users. 1 test was positive for Buprenorphine that confirmed collected medical information.

*Nodewise eigenvector centrality*

Supplemental Figure 3 shows the nodewise eigenvector centrality (EC) based on the F-statistics from the edgewise group comparisons. High values indicate a relatively strong cumulative group effect independent of the significance threshold applied in the edgewise analysis. Highest EC values are seen for the DAN and the SFG/IFG/ACC nodes.


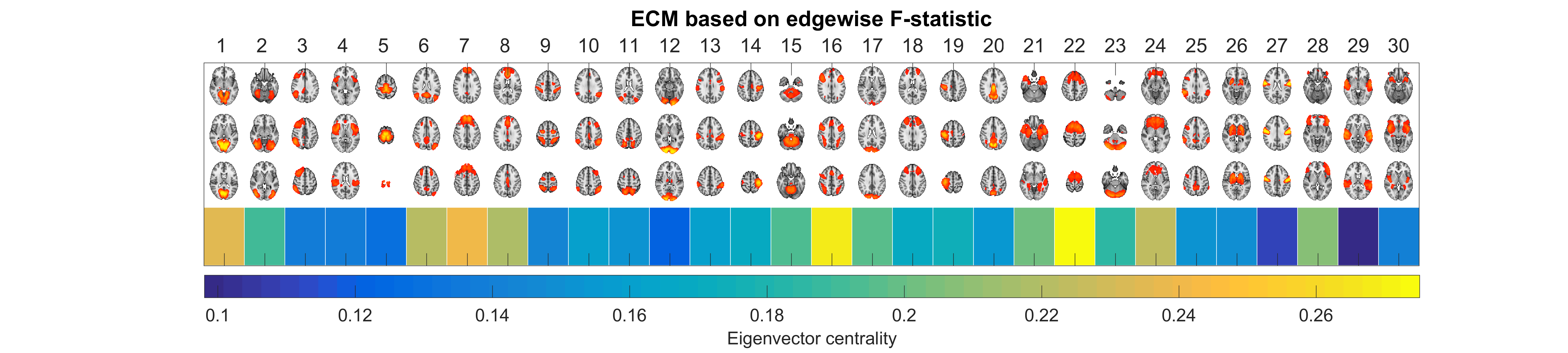
**Supplemental Figure 3.** Nodewise EC based on F-statistics from edgewise group comparisons.

*Correlations with T/E ratio across the full sample*


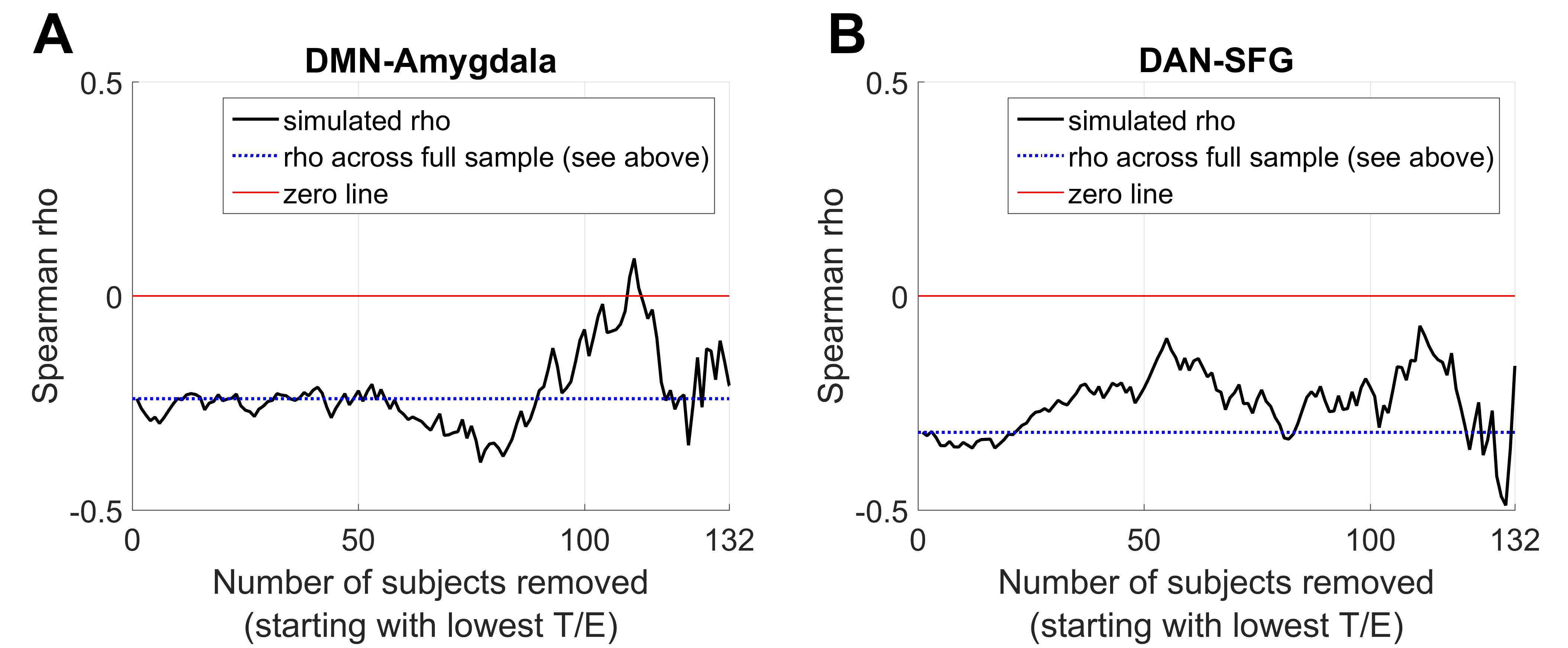
Supplementary Figure 4 shows the correlation between edge connectivity and T/E ratio across the full range of T/E ratios obtained by first running the analysis across all subjects and then recomputing correlations when excluding one subject after another, starting with the lowest T/E ratio. Correlation coefficients are negative across the T/E range, indicating that associations are not simply recapitulating the group differences.

**Supplemental Figure 4.** Correlation between edge connectivity and T/E ratio by T/E ratio.

*Group effects on edge connectivity when including various confounders in the models*

Supplemental Table 1 shows the relevant statistics from group comparisons when including different clinical and demographic covariates in the ANCOVAs. Briefly, the group effects obtained in the main analysis remained in all models.

|  | **DMN-amygdala** | | |  | | | **DAN-SFG** | | |  | | |
| --- | --- | --- | --- | --- | --- | --- | --- | --- | --- | --- | --- | --- |
| *Covariate*  */Subsample* | F_group_ | *P*_group_ | P eta_group_ | | Post hoc | F_group_ | | P eta_group_ | *P*_group_ | | Post hoc |  |
| Age (n=125) | 9.96 | <.001 | 0.14 | | a***, b** | 10.17 | | 0.14 | <.001 | | a***, b* |  |
| VIQ (n=125) | 10.82 | <.001 | 0.15 | | a***, b** | 9.14 | | 0.13 | <.001 | | a***, b* |  |
| Weekly Alcohol (n=123) | 8.59 | <.001 | 0.13 | | a**, b** | 10.26 | | 0.15 | <.001 | | a***, b* |  |
| ASR Drugs (n=107) | 8.15 | .001 | 0.14 | | a***, b* | 5.84 | | 0.10 | .004 | | a** |  |
| ASR Anxious/Depressed (n=107) | 7.91 | .001 | 0.13 | | a**, b* | 8.12 | | 0.14 | .001 | | a*** |  |
| ASR Attention  Problems (n=109) | **9.18** | <.001 | 0.15 | | a***, b* | **9.72** | | 0.16 | .002 | | a*** |  |
| Total Problems (n=106) | 7.97 | .001 | 0.14 | | a***, b* | **10.47** | | 0.17 | <.001 | | a*** |  |
| Narcotics in urine  excluded (n=102) | 9.50 | <.001 | 0.16 | | a***, b** | 7.80 | | 0.14 | 0.001 | | a***, b* |  |

**Supplemental Table 4. G**roup differences in edge connectivity controlling for potential confounders. GLMs were performed for the edges showing significant main effects of group to control for potential confounders, by including them as covariates in the GLMs. An additional analysis was carried out with participants showing traces of narcotics in the urine excluded, to reassure that the group differences not could be ascribed the use of narcotics. *n* denotes the number of participants in each sub-analysis. Abbreviations: p eta: partial eta (explained variance by group), VIQ: Verbal-IQ; ASR: Adult Self-Report. Bonferroni Post Hoc test a = controls significantly different from current users, b = current users significantly different from previous users. All effects were in the form of reduced connectivity in current AAS users compared to the other groups. Bold F value refers to significant unique association between the relevant ASR scale and edge connectivity.

**References**

1999. Wechsler Abbreviated Scale of Intelligence. PsychCorp, Harcourt Assessment, Inc, San Antonio, TX, USA.

Achenbach, T.M., 1990/1997. Young Adult Self Report. University of Vermont, Department of Psychiatry, Burlington, VT.

Bjørnebekk, A., Walhovd, K.B., Jørstad, M., Due-Tonnessen, P., Hullstein, I., Fjell, A.M., 2016. Structural Brain Imaging of Long Term Anabolic-Androgenic Steroid Users and Non- using Weightlifters. Biol Psychiatry.

First, M.B., Spitzer, R.L., Gibbon, M., Williams, J.B.W., 1996. Structural Clinical INterview for DSM-IV Axis I Disorders, Clinical Trials Version (SCID-CV). American Psychiatric Press, Inc., Washington, D.C.

Kanayama, G., Brower, K.J., Wood, R.I., Hudson, J.I., Pope, H.G., Jr., 2009. Issues for DSM-V: clarifying the diagnostic criteria for anabolic-androgenic steroid dependence. Am J Psychiatry 166, 642-645.

Pope, H.G., Jr., Katz, D.L., 1994. Psychiatric and medical effects of anabolic-androgenic steroid use. A controlled study of 160 athletes. Arch Gen Psychiatry 51, 375-382.

Pope, H.G., Kean, J., Nash, A., Kanayama, G., Samuel, D.B., Bickel, W.K., Hudson, J.I., 2010. A diagnostic interview module for anabolic-androgenic steroid dependence: preliminary evidence of reliability and validity. Exp Clin Psychopharmacol 18, 203-213.

Pruim, R.H., Mennes, M., Buitelaar, J.K., Beckmann, C.F., 2015. Evaluation of ICA-AROMA and alternative strategies for motion artifact removal in resting state fMRI. Neuroimage 112, 278-287.

Roalf, D.R., Quarmley, M., Elliott, M.A., Satterthwaite, T.D., Vandekar, S.N., Ruparel, K., Gennatas, E.D., Calkins, M.E., Moore, T.M., Hopson, R., Prabhakaran, K., Jackson, C.T., Verma, R., Hakonarson, H., Gur, R.C., Gur, R.E., 2016. The impact of quality assurance assessment on diffusion tensor imaging outcomes in a large-scale population-based cohort. Neuroimage 125, 903-919.

Smith, S.M., Miller, K.L., Salimi-Khorshidi, G., Webster, M., Beckmann, C.F., Nichols, T.E., Ramsey, J.D., Woolrich, M.W., 2011. Network modelling methods for FMRI. Neuroimage 54, 875-891.
